# Reciprocal Interference of Experimental Dyslipidemia and Food Allergy in the Evolution of Both Diseases

**DOI:** 10.1155/2013/545184

**Published:** 2013-06-06

**Authors:** A. C. Gomes-Santos, J. L. Gonçalves, T. R. Fonseca, A. R. Marques, L. P. A. Dourado, D. C. Cara, J. I. Alvarez-Leite

**Affiliations:** ^1^Department of Food, Faculty of Pharmacy, Universidade Federal de Minas Gerais, Belo Horizonte, MG, Brazil; ^2^Department of Biochemistry and Immunology, Institute of Biological Sciences, Universidade Federal de Minas Gerais, Avenida Antônio Carlos 6627, Pampulha, 30161-970 Belo Horizonte, MG, Brazil; ^3^Department of Morphology, Institute of Biological Sciences, Universidade Federal de Minas Gerais, Belo Horizonte, MG, Brazil

## Abstract

*Background*. Food allergies have been shown to reduce serum triacylglycerol, glucose, cholesterol, and free fatty acid levels in mice. In turn, dyslipidemias, especially dyslipidemias presenting with low levels of HDL cholesterol, are important risk factors for the development of atherosclerosis. However, the consequences of food allergies on dyslipidemia and atherosclerosis have not been fully investigated. *Methods*. Food allergy was induced using an egg white solution (EWS) in ovalbumin- (OVA-) sensitized C57BL/6 and low-density lipoprotein receptor knockout mice (LDLr^−/−^) for 5 weeks and was confirmed by the high production of anti-OVA IgE and IgG1 antibodies in both mouse strains. *Results*. The allergic C57BL/6 mice exhibited EWS aversion that was associated with less visceral fat and high levels of anti-Ova IgE antibodies after 5 weeks of EWS intake compared to controls. However, LDLr^−/−^ allergic mice showed reduced anti-Ova IgE levels that were similar to the nonsensitized group. The LDLr^−/−^ allergic mice also demonstrated a reversal of food aversion and sustained visceral fat after 5 weeks of allergy. Although HDL cholesterol levels were reduced in both sensitized mouse strains, lipid deposition in thoracic and abdominal aorta as well as area and composition of atherosclerotic plaques as unaffected by chronic ingestion of EWS. *Conclusion*. LDLr^−/−^ mice develop an attenuated food allergy, as they showed a reversal of food aversion and lower IgE production after 5 weeks of induced allergy. The development of atherosclerosis, in turn, was not accelerated in the allergic LDLr^−/−^ group despite the more atherogenic lipid profile.

## 1. Introduction

Under physiological conditions, interactions of gut mucosa and dietary proteins are associated with induction of oral tolerance. It has been reported that 130–190 g of protein is absorbed in small intestine daily. Indeed, lack of homeostasis of these immunological activities can cause disorders such as inflammatory bowel disease and food allergies [1, 2]. 

Food allergies develop because of an adverse immune response to food proteins. IgE-mediated food allergies (type I food allergies) account for the majority of food allergic reactions, and the onset of symptoms can occur immediately after ingestion of the allergen [3, 4]. Food allergies are particularly frequent among children, and the symptoms include vomiting, weight loss, abdominal pain, malabsorption, atopic eczema, urticarial, and angioedema [5]. Epidemiological studies have suggested that there has been a significant increase in the prevalence of allergic disease in the past two or three decades [6].

Our research group has developed an experimental model of food allergy in which Ova-sensitized mice receive EWS as their only liquid source. This model simulates several aspects of food allergies in humans, such as increased anti-Ova IgE/IgG1 antibody levels in the serum, intestinal edema, eosinophil infiltration, and weight loss [7]. Moreover, the model has shown a correlation between mice that have an aversion to antigen and the production of IL-4 and Ova-specific antibodies [8]. 

Lipid metabolism is altered during acute and chronic inflammatory responses [9]. Weight loss and metabolic changes have been shown to occur during food allergic responses and inflammatory bowel disease [10, 11]. Previous studies by our group have indicated that allergic mice show low serum concentrations of triacylglycerol, glucose, total cholesterol, and free fatty acids [8]. Alterations in serum lipids in allergic individuals could be a risk factor for the development of atherosclerosis. Pesonen et al. [12] have reported that serum cholesterol levels in infancy are inversely associated with the development of allergies in children. In addition, Schäfer et al. have found that HDL cholesterol levels are associated with atopy in adults [13].

LDLr^−/−^ mice have been used as a well-established model of dyslipidemia and atherosclerosis. These mice exhibit hypercholesterolemia due to the accumulation of LDL and develop modest atherosclerotic lesions in the proximal aortic root when they are fed a Western diet for a few weeks [14]. In contrast, C57BL/6 wild type mice do not develop atherosclerotic lesions even when fed a Western diet. Because metabolism plays a role in the manifestation of allergies, our study aimed to investigate the effect of food allergy on lipid profile and atherosclerotic disease; we also investigated the reciprocal effect of dyslipidemia on allergy development using LDLr^−/−^ atherosclerosis-susceptible mice versus C57BL/6 control mice.

## 2. Methods

### 2.1. Animals and Diet

Twenty-four twelve-week-old female LDLr^−/−^ mice and twenty-four C57BL/6 atherosclerosis-resistant mice (control) were used. Mice were kept in the animal facility of the Atherosclerosis and Nutritional Biochemistry Laboratory (ICB/UFMG) and fed a cholesterol-rich diet (AIN-93M standard diet [15] containing 1.25% cholesterol). After accounting for initial body weight and total cholesterol levels, mice were distributed into the following four groups: WT OVA^−^: wild type C57BL/6 nonsensitized mice; WT OVA^+^: wild type C57BL/6 mice sensitized with ovalbumin; LDLr^−/−^ OVA^−^: non-sensitized mice; and LDLr^−/−^ OVA^+^: mice sensitized with ovalbumin.

Sensitization Protocol: twenty-one days prior to the beginning of the experiment, mice from OVA^+^ groups received a subcutaneous (sc) injection of 0.2 mL of saline containing 10 *μ*g of Ova (5 times crystallized hen's egg albumin) plus 1 mg of Al(OH)_3_ as an adjuvant and those from OVA^−^ (Control) groups received 0.2 mL saline (0.9%) with adjuvant [1 mg Al(OH)_3_]. Secondary sensitization consisted of a sc injection of 10 *μ*g of soluble OVA 14 days after the primary sensitization. OVA- groups received the same volume of saline at the same day as proposed by Saldanha et al. [7].

Experimental Design: at the beginning of experiment (seven days after the secondary sensitization), water was replaced by 20% egg white solution (EWS) containing approximately 10 mg of Ova/mL for a period of 5 weeks (from day 0 to day 35). At the fifth experimental week animals were euthanized after overnight fasting for blood and organs collection.

Food intake and body weight were evaluated daily and weekly, respectively. The experimental protocol was approved by the Animal Care Committee of Universidade Federal de Minas Gerais (UFMG, CETEA no. 42/2007).

### 2.2. Blood, Liver, and Cecal Samples

Blood samples were collected on day 0 and on day 35 of the experiment. Serum HDL cholesterol (HDLc) levels were measured after separation using a commercially available kit. HDLc, total cholesterol, and triacylglycerol levels in blood and tissues were determined using commercially available kits. The non-HDLc fraction was calculated as the difference between the total cholesterol and HDLc fractions. Livers were removed and frozen at −80°C. Hepatic and cecal lipids were extracted as previously described [16].

### 2.3. Determination of Anti-Ova IgE and IgG1 Antibodies in the Serum

The ELISA for IgG1 was carried out on plates coated with Ova, 100 *μ*L of a 1 : 1 600 dilution of mouse sera, and biotinylated goat anti-mouse IgG1. The reactions were developed with the streptavidin-peroxidase conjugate, o-phenylenediamine, and H_2_O_2_. The plates were read at 492 nm on an automated ELISA reader. Anti-Ova IgE antibodies were measured by a capture ELISA on plates coated with rat anti-mouse IgE, 50 *μ*L of total serum, and biotinylated Ova as previously described [17]. The results are reported as arbitrary units using a positive reference serum that was assigned 1 000 units.

### 2.4. Histological Analysis

The heart and proximal sections of the aorta were removed and cleaned of adventitial tissue. The top halves of the hearts were obtained using a stereoscope and fixed by immersion in 4% paraformaldehyde in 0.1 M PBS room temperature. The specimens were routinely processed for paraffin embedding and analyzed as previously described [18] with slight modifications. Briefly, consecutive sections (6-*μ*m thick) throughout the aortic root area (300 *μ*m) were stained with hematoxylin and eosin. The aortic root area was determined by the proximal presence of aortic valve leaflets. One out of every five sections (from 10 sections per mouse) was kept for morphometric analysis with an image analyzer to process images obtained from a microscope coupled to a digital camera. The total lesion area from each animal represented the sum of the lesion areas obtained from the 10 selected sections. The results were expressed as the average of the total lesion area per group. To determine the cellularity of the lesion, the number of inflammatory cells per field was counted, taking into consideration their morphological aspect. The area of each field was determined automatically by Image-Pro Plus software (from 5 mice with 3 nonconsecutive sections per animal). Extracellular collagen was visualized using Gomori's trichrome stain [18, 19] and area determined automatically by Image-Pro Plus software. The results were given as percentage of collagen inside lesion area. All microscopic analyses were performed by a blinded operator. Thoracic and abdominal portions of aortas were cleaned of adventitia and stained with Sudan IV before measurement of lesion area using an image analyzer.

### 2.5. Statistical Analysis

 Data were analyzed using one-way analysis of variance (ANOVA) or Student's *t*-test where appropriate. The level of significance was set at *P* < 0.05. If ANOVA revealed differences, the groups were compared by the Tukey's test.

## 3. Results

Food intake and final body weight were not affected by the chronic food allergy induced in C57BL/6 and LDLr^−/−^ mice. However, there was a reduction in the relative weight of visceral fat in the C57BL/6 OVA^+^ mice that was not observed in the LDLr^−/−^ OVA^+^ mice (Table 1). 

**Figure 1:**
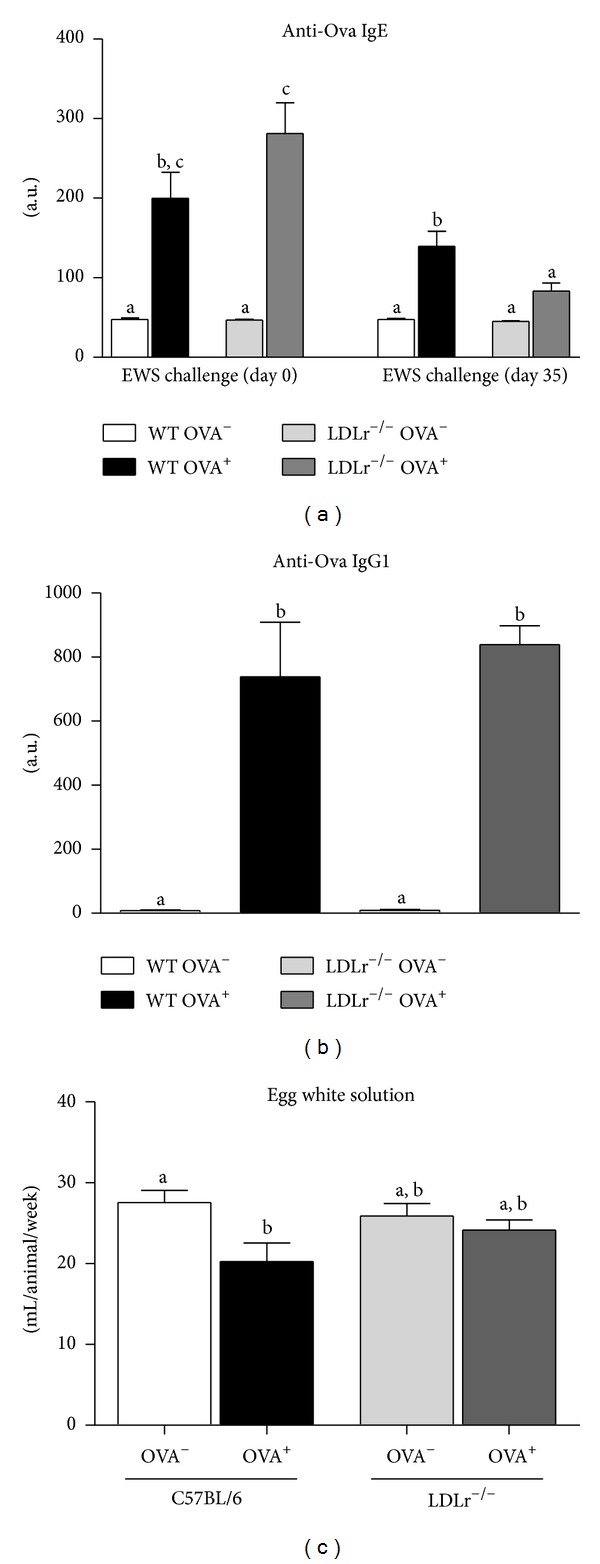
Serum anti-Ova IgE (a) and IgG1 (b) antibody response of C57BL/6 (WT) or LDLr^−/−^ mice nonsensitized (OVA^−^) or sensitized (OVA^+^) with 10 *μ*g of Ova at the beginning and 14th experimental day and receiving 20% egg white solution (EWS) from 21st to 35th experimental days. Weekly, EWS intake is present in (c). Bars represent means and vertical lines represent SEM. *N* = 6 mice/group. Data showing different letters in the same day are statistically different (*P* < 0.05).

Control (OVA^−^) mice of both strains had the same levels of anti-Ova IgE antibodies in the serum on day 0, suggesting that the sensitization procedure was consistent between the strains. However, after 5 weeks of oral EWS challenge, although C57BL/6 OVA^+^ mice had higher levels of anti-Ova IgE antibodies when compared with C57BL/6 OVA^−^ mice, allergic LDLr^−/−^ OVA^+^ mice showed similar levels of anti-Ova IgE antibodies when compared with nonsensitized LDLr^−/−^ OVA^−^ mice (Figure 1(a)). In contrast, anti-Ova IgG1 levels were increased in allergic mice but were not altered between the C57BL/6 and LDLr^−/−^ mice (Figure 1(b)). Aversion to the egg white solution, which was measured as the EWS consumption per week, was observed in allergic C57BL/6 and LDLr^−/−^ mice in the first week of consumption. However, EWS aversion was reversed in LDLr^−/−^ OVA^+^ mice by the end of the experimental period (Figure 1(c)). 

**Figure 2:**
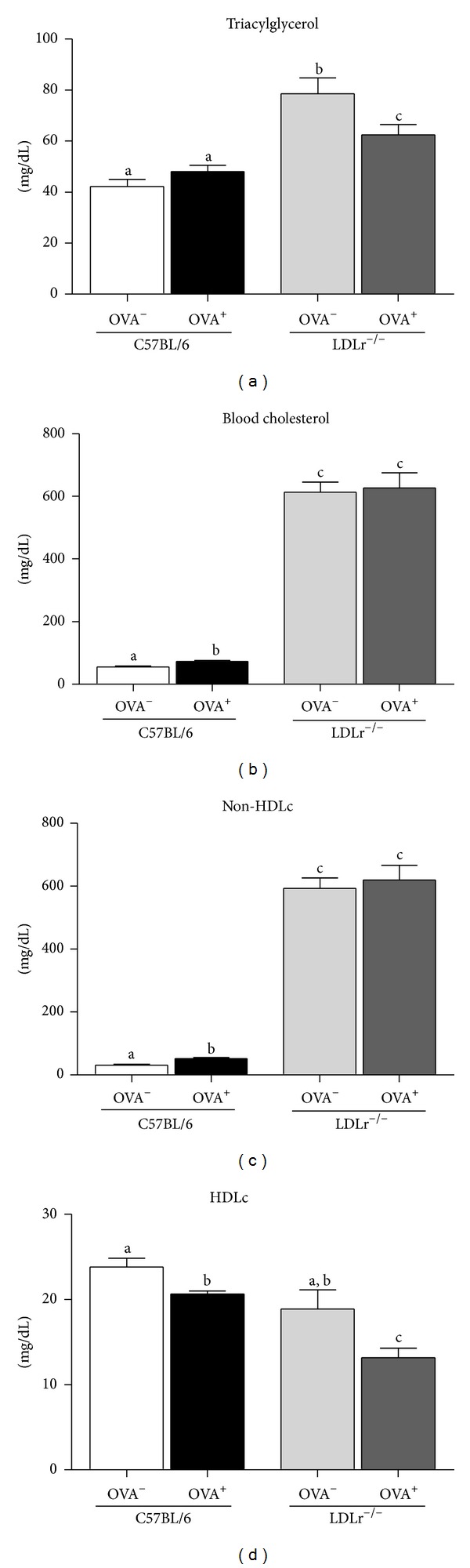
Serum lipid profile of C57BL/6 (WT) or LDLr^−/−^ mice nonsensitized (OVA^−^) or sensitized (OVA^+^) with 10 *μ*g of Ova at the beginning and 14th experimental day and receiving 20% egg white solution from 21st to 35th experimental days. Bars represent means and vertical lines represent SEM. *N* = 6 mice/group. Data showing different letters in same animal type are statistically different (*P* < 0.05). Non-HDL cholesterol levels were calculated as the difference between total and HDL-cholesterol concentrations.

The triacylglycerol (TAG) concentrations were higher in LDLr^−/−^ compared to C57BL/6 mice. However, food allergy reduced TAG concentrations only in LDLr^−/−^ mice (Figure 2(a)). Total serum cholesterol levels increased in sensitized C57BL/6 mice (Figure 2(b)) due to the reduction in HDLc and subsequent increase in non-HDLc fractions (Figures 2(c) and 2(d)). Although total cholesterol levels were not affected by allergy, LDLr^−/−^ mice showed a reduction in HDLc levels (Figure 2(b)). These lipoprotein profiles led to a higher total cholesterol/HDLc ratio in both OVA^+^ groups compared to controls (data not shown). Because neither hepatic and cecal lipids nor cholesterol levels in sensitized C57BL/6 and LDLr^−/−^ mice changed (Table 2), the alterations in the lipid profile were not due to changes in lipid concentrations in the liver or fecal matter, as they were similar among the groups.

**Figure 3:**
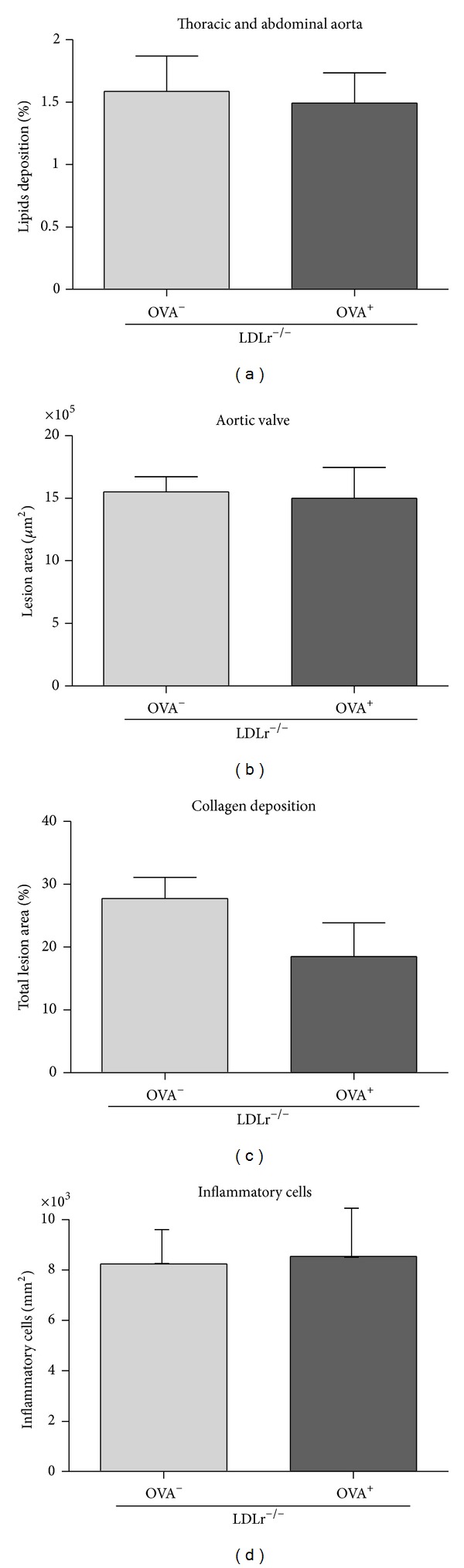
Atherosclerosis area in aorta (a); lesion area in aortic root (b) and percentage of collagen deposition (c); and inflammatory cells infiltration in lesion area of aortic root (d) of LDLr^−/−^ mice nonsensitized (OVA^−^) or sensitized (OVA^+^) with 10 *μ*g of Ova at the beginning and 14th experimental day and receiving 20% egg white solution from 21st to 35th experimental days. Bars represent means and vertical lines represent SEM. *N* = 11 mice/group. No statistical differences were detected (*P* > 0.05, Student's *t*-test).

As C57BL/6 wild type mice do not develop atherosclerotic lesions spontaneously, we evaluated the atherosclerotic process only in LDLr^−/−^ mice. Interestingly, lesion area, total inflammatory cell infiltration, and collagen deposition in the aortic valve were not affected by induction of food allergy (Figures 3(a)–3(d)), despite the alterations in blood lipid levels.

## 4. Discussion

In the present study, we showed that the development of food allergy, due to a Th2 immune response, is attenuated in the atherosclerosis-susceptible LDLr^−/−^ mice, which mount a Th1 response when fed a cholesterol-rich diet [20]. To the best of our knowledge, this is the first study to address the influence of atherosclerosis susceptibility on the development of food allergy.

The clinical manifestation of egg white allergy has already been described in C57BL/6 mice by our group, and it is characterized by a loss of visceral fat during the induction of allergy [8]. When the two OVA^−^ groups were compared, LDLr^−/−^ mice had less abdominal fat than the wild type controls. However, LDLr^−/−^ OVA^+^ mice maintained visceral fat after food allergy induction when compared with C57BL/6 OVA^+^ mice, suggesting that the effects of food allergy were attenuated in LDLr^−/−^ mice. These data were reinforced upon measurement of EWS aversion in LDLr^−/−^ mice. The EWS aversion was also attenuated in LDLr^−/−^ OVA^+^ mice after five experimental weeks when compared with the control mice. Moreover, IgE antibody levels, which represent a known marker of allergy, returned to basal levels only in LDLr^−/−^ OVA^+^ mice. The connection between allergenic proteins and increased levels of IgE antibodies has been previously described [21]. Basso et al. (2003) showed that treatment with anti-IgE antibodies 7 days prior to oral challenge prevented EWS aversion in Ova-sensitized BALB/c mice [22]. Taken together, our results suggest that food allergy is attenuated in LDLr^−/−^ mice compared to control mice.

Genetic predisposition is a factor that directly affects the allergic response [23]. BALB/c mice have been frequently used as a model of food allergy due to their high production of IgE antibodies [24]. In contrast, C57BL/6 mice are considered more resistant to sensitization with antigen [25]. LDLr^−/−^ mice, which are on a C57BL/6 background, display a predominant Th1 response with an important role for proinflammatory cytokines, such as TNF-*α* and IFN-*γ* that accelerate the atherosclerotic process [19, 26]. A cholesterol-rich diet exacerbates the Th1 response and, consequently, the progression of the atherosclerotic lesion. We suggest that the Th1 genetic profile in conjunction with the consumption of a cholesterol-rich diet reduced the Th2 immune response that is characteristic of food allergy, thereby limiting or attenuating its clinical signs in LDLr^−/−^ mice.

The main alteration of the lipid profile observed in our study was the reduction in HDL cholesterol levels in both OVA^+^ groups. Changes in lipid metabolism after allergy induction have already been described by Dourado et al. [8] and are related to metabolic and immunological responses that are linked to immediate hypersensitivity. The reduction in HDLc levels is associated with the acute-phase response triggered by inflammatory processes, such as allergy. At this acute stage, there are usually increased circulating levels of triacylglycerols and decreased levels of HDLc. The reduction in HDL levels in both allergic C57BL/6 and LDLr^−/−^ mice could be due to increased levels of cytokines that reduce ABCA1 gene expression by inhibiting cholesterol efflux [27]. The decreased levels of HDLc may also be related to increased levels of histamine. Liao et al. (1997) have shown that both endogenous and exogenous histamines reduce the levels of HDLc and the expression of the LDL receptor in the liver, both of which play an important role in regulating serum lipoprotein balance [28]. 

Although the characteristic Th1 profile of LDLr^−/−^ mice attenuated the severity of food allergy, the development of atherosclerosis was not influenced by food allergy, as no differences were found in the lesion area of the aortic valve and aorta or in the collagen content or inflammatory cell infiltration in the aortic valve. It might be assumed that the increased atherogenic (cholesterol/HDLc) ratio, due to the high levels of total cholesterol and the low levels of HDLc, would accelerate the development of atherosclerosis in LDLr^−/−^ OVA^+^ mice. However, we did not find any differences in lesion area, lesion vulnerability (measured as collagen content), or lesion inflammatory cell infiltration between LDLr^−/−^ groups. Because pro-inflammatory cytokines, such as IFN-*γ* and TNF-*α*, are important inducers of foam cell formation and fibrous cap degradation (via overexpression of the metalloprotein MMP9), the moderate Th2 response induced by food allergy could prevent the inflammatory component of atherosclerosis and impair the development of more advanced atherosclerotic lesions even in the presence of high levels of cholesterol.

In conclusion, our results suggest that a food allergy can be induced in atherosclerosis-susceptible LDLr^−/−^ mice. However, these mice develop an attenuated food allergy with a reversal of the food aversion and lower IgE antibody production after 5 weeks of allergy evolution. The development of atherosclerosis, in turn, is not accelerated in LDLr^−/−^ OVA^+^ mice despite the more atherogenic lipid profile, which is a consequence of ovalbumin sensitization.

## Figures and Tables

**Table 1:** Food intake, final body weight, and percentage of weight that was visceral fat of C57BL/6 (WT) or LDLr^−/−^ mice nonsensitized (OVA^−^) or sensitized (OVA^+^) with 10 *μ*g of Ova at the beginning and 14th experimental day and receiving 20% egg white solution from 21st to 35th experimental days.

Parameter	C57BL/6		LDLr^−/−^	
	OVA^−^	OVA^+^	OVA^−^	OVA^+^
Food intake (g/mice/week)	20.2 (0.7)^a^	20.2 (0.6)^a^	17.7 (0.7)^a^	17.7 (0.8)^a^
Final body weight (g)	25.3 (0.6)^a^	25.7 (0.8)^a^	22.6 (0.3)^b^	22.2 (0.2)^b^
% body weight as visceral fat	2.2 (0.15)^a^	1.7 (0.21)^b^	1.3 (0.16)^b^	1.2 (0.13)^b^

Results are expressed as the mean (SE). *n* = 11/group. Data showing different letters in the same row are statistically different (*P* < 0.05).

**Table 2:** Hepatic and cecal lipids of C57BL/6 (WT) or LDLr^−/−^ mice nonsensitized (OVA^−^) or sensitized (OVA^+^) with 10 *μ*g of Ova at the beginning and 14th experimental day and receiving 20% egg white solution from 21st to 35th experimental days.

	C57BL/6		LDLr^−/−^	
	OVA^−^	OVA^+^	OVA^−^	OVA^+^
Liver				

Total lipids (mg/g)	126 (15)	141 (9)	123 (7)	123 (9)
Cholesterol (mg/g)	6.4 (1.1)	7.4 (1.0)	7.1 (1.0)	7.0 (1.1)

Cecum				

Total lipids (mg/g)	39.0 (5.4)	38.7 (9.5)	42.8 (10.1)	22.5 (4.5)
3-*α*-OH steroids (mg/g)	4.1 (0.4)	4.5 (0.5)	5.3 (0.8)	4.8 (0.6)

No significant differences were observed between OVA^−^ and OVA^+ ^groups in the same strain (*n* = 11/group).
